# Limitations in clinical outcome after posterior stabilization of thoracolumbar fractures do not correlate with dynamic trunk muscle dysfunction: an ultrasound controlled prospective cohort study

**DOI:** 10.1186/s40001-018-0323-z

**Published:** 2018-05-24

**Authors:** Miguel Pishnamaz, Ulrike Schemmann, Christian Herren, Klemens Horst, Frank Hildebrand, Philipp Kobbe, Hans-Christoph Pape

**Affiliations:** 1 Department of Orthopaedic Trauma, University of Aachen Medical Center, Pauwelsstrasse 30, 52074 Aachen, Germany; 2 Division of Physical Therapy, University of Aachen Medical Center, Pauwelsstrasse 30, 52074 Aachen, Germany; 3 Department of Trauma, University of Zurich, Rämistrasse 100, 8091 Zurich, Switzerland

**Keywords:** Percutaneous spinal stabilization, Thoracolumbar fracture, Outcome, Ultrasound, Muscular changes, Lumbar multifidus, Transverse abdominis muscle

## Abstract

**Background and Purpose:**

Posterior stabilization of the spine is associated with iatrogenic muscle damage. This is discussed to represent an important cause of postoperative pain, especially in open reduction and fixation. The aim of this study was to visualize muscular changes after open or percutaneous posterior stabilization of traumatic thoracolumbar spine fractures and to investigate whether or not these changes are related to the clinical outcome.

**Methods:**

This prospective cohort study was performed between 05/2012 and 10/2014. A group of posteriorly stabilized patients (study group; SG) with traumatic fractures (AOSpine Type A3 or A4) of the thoracolumbar junction (T11–L2) without neurological deficit were matched to a healthy control group (CG) by age, gender and body mass index. Follow-up: 12 months after surgery. Parameters: muscle size, voluntary muscular activation (VMA) using a standardized ultrasound protocol and standardized questionnaires (VAS Spine Score; ODI; SF-36) were analyzed. Statistics: SPSS (Version 20, 76 Chicago, IL, USA). *T* test, Chi squared test, analysis of variance and a correlation analysis were performed. Significance level was at *p* < 0.05.

**Results:**

Twenty-five patients (SG) and 23 control individuals (CG) were included. At follow-up, voluntary muscular activation of the lumbar multifidus (LM) as well as the transverse abdominis muscle (TrA) was diminished in all patients compared to the control group (VMA LM at level L3/4: SG 3.2%; CG 5.1%; *p* < 0.05; VMA TrA: SG 33.43%; CG 37.84%; *p* < 0.05). Concomitant interviews revealed health restrictions in all patients when compared with the control group. A correlation between muscle function and clinical outcome could not been demonstrated (rs > 0.07; NS).

**Conclusion:**

In surgically treated A3 and A4 fractures, there is continuous muscular deficit 1 year after surgery as documented by ultrasound and clinical control. But, by means of our study we conclude that those muscular deficits alone seem not to be decisive for the clinical outcome 1 year after surgery.

## Background

Posterior stabilization is an established treatment option of unstable fractures of the thoracolumbar junction. Iatrogenic muscle damage during muscle retraction and screw placement is unavoidable. The literature suggests that percutaneous stabilization is associated with less iatrogenic muscle trauma when compared with open fixation [1, 2], but to date it is unclear whether this affects the clinical outcome. The lumbar multifidus (LM) and the transversus abdominis muscle (TRA) play an important role in spine stabilization [3–6]. The transversus abdominis muscle is the only muscle that is constantly attached to the thoracolumbar fascia and therefore named as “musculofascial corset” of the lumbar spine [7]. It is discussed that deficits of this muscle may result in low back pain [8].

The multilayered multifidus muscle stabilizes mobile segments of the spine by applying adjusted muscle tension and concomitant compression to the spinal facet joints [9–12]. The muscle is sectionally innervated by the posterior branch of the spinal nerves. Studies on degenerative diseases showed neurogenic and myogenic changes in the LM postoperatively [13, 14]. Furthermore, open posterior stabilization causes denervation of these muscles [1, 2, 15–17] and several studies attribute postoperative pain, limited activities of daily living and diminished life quality to these muscular changes [13, 18, 19].

The purpose of this clinical follow-up study was twofold: firstly to visualize muscular changes and muscular function following either open or percutaneous posterior stabilization of traumatic thoracolumbar spine fractures and, secondly, to investigate whether or not these changes are related with clinical outcome.

## Methods

### Ethics

This study was performed in accordance with the ethical standards of the responsible committee on human experimentation and with the Helsinki Declaration of 1975, as revised in 2000.

### Study design

This study was conducted as a prospective single-center cohort study between May 1, 2012 and October 31, 2014. Inclusion criteria: patients aged between 18 and 80 years at the time of injury, treated by bisegmental posterior stabilization of an acute burst fracture (AOSpine Type A3 or A4) of the thoracolumbar junction (T11–L2). Exclusion criteria: patients with neurological deficits, multi-level spine trauma, pregnancy or polytrauma (ISS; NISS ≥ 16).

A written consent form was obtained from all participants prior to the study.

### Group description

The participants were divided into two main groups:

1. Group surgery (SG; study group): The group comprised patients who had undergone bisegmental posterior stabilization of an acute fracture of the thoracolumbar junction.
2. Group control (CG): a group of healthy participants without pre-existing back pain was matched to SG regarding age, BMI and gender distribution.

The group surgery was further divided into two subgroups:

1. Group open surgery (OS): The surgical approach was performed by the conventional median technique. In this case the paravertebral muscle was detached by diathermal preparation and retracted by means of hooks. Within this procedure, pedicle screw placement was performed under visual control. Consequently, the longitudinal rods were inserted and fixed in a top loading position.
2. Group minimally invasive surgery (MIS): The surgery was performed percutaneously. In this case, the entry point to the pedicle was determined by fluoroscopy and a 2.5 cm skin incision was set slightly lateral to the pedicle entrance point. After longitudinal incision of the fascia, the muscles were bluntly dissected with the fingertip. Once screw placement was completed, the longitudinal rods were inserted cranially through additional incisions.

Initially, all patients were treated using a stand-alone posterior fixation without a posterior spondylodesis. Depending on the fracture morphology anterior fusion was performed in the early postoperative course.

All patients received standard of care treatment. The decision for open or percutaneous approach was made by the surgeon’s experience. It was not related to fracture type or any other objective criteria.

### Follow-up

After 12 months of postoperative follow-up, patients underwent a standardized ultrasound examination performed by a well-trained physician. This examination was carried out in exactly the same way to the control group. In addition, all patients and control were subjected to standardized questionnaires.

### Ultrasound examination

Ultrasound was performed by the usage of the Siemens ACUSON 2000 ultrasound unit (Siemens AG Medical Solutions Germany). Examination of the multifidi muscles was performed by the use of a 9 MHz linear ultrasound probe, whereas the abdominal muscles were examined by a 4.5 MHz convex probe. All ultrasound examinations were performed using the b-mode. Patient positioning and examination was standardized in accordance to previous studies [20, 21]. To avoid disturbing interference of the internal fixator to the ultrasound visualization, the examination of the multifidi muscles was performed below the fracture site in the spinal segments L3/4 and L4/5.

**Fig. 1:**
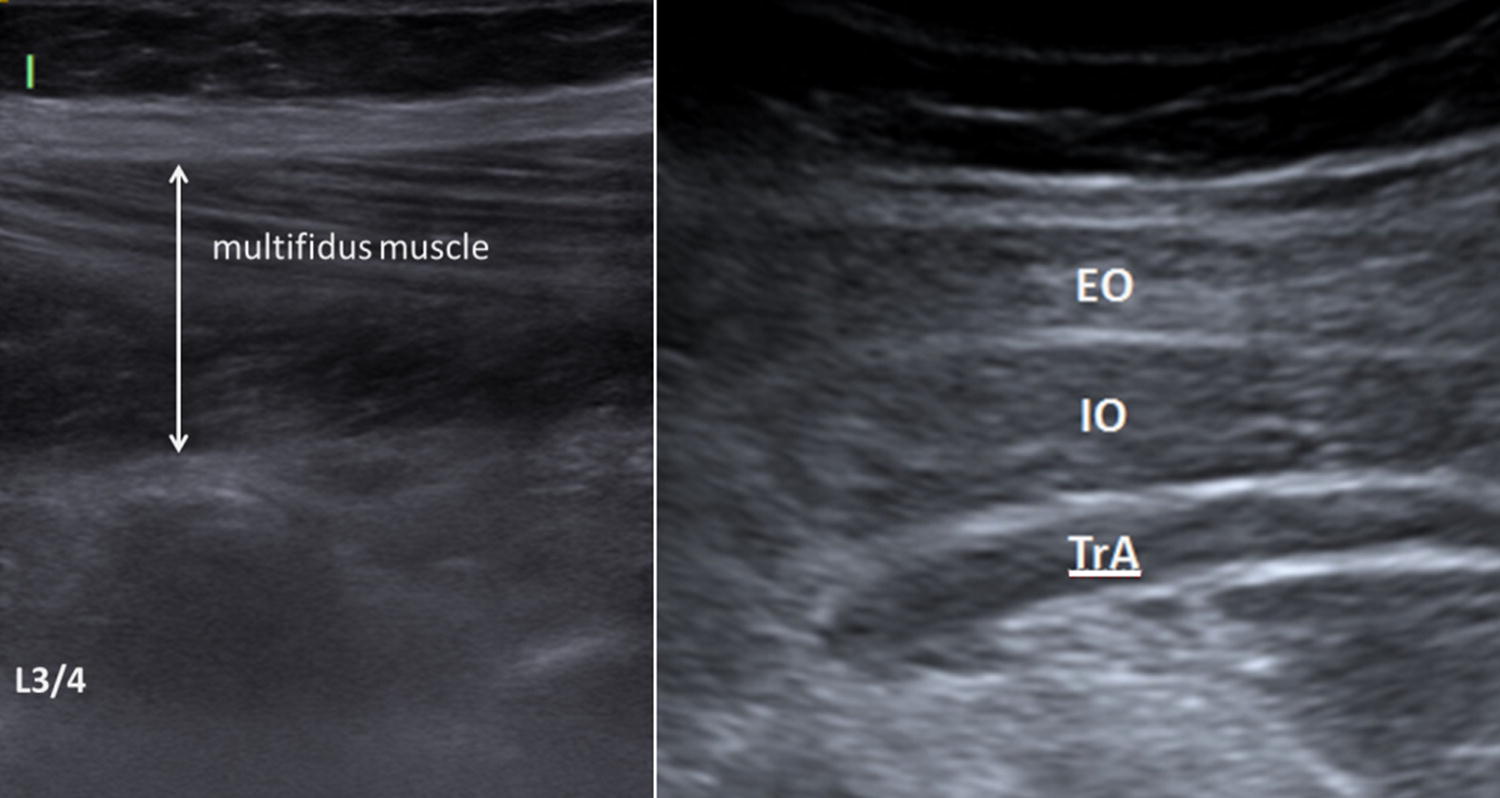
Example of an ultrasound examination: left side: illustration of the lumbar multifidus muscle at level L3/4; right side: illustration of the abdominal muscles (*EO* external oblique muscle; *IO* internal oblique muscle; *TrA* transversus abdominis muscle)

The muscular diameter of the oblique abdominal muscles, the transversus abdominis and the multifidi muscles were measured in a contracted and in a relaxed muscle state. The muscle function was measured as the percentage change of the muscle thickness during contraction and relaxation (VMA voluntary muscular activity) (Fig. 1).

### Functional outcome scores

During the follow-up examination, three different questionnaires (VAS Spine Score, the Oswestry Disability Index and the SF-36) were obtained.

The VAS spine score is focused on the pain level in different life situations [22].

The Oswestry Disability Index (ODI) is a disease-specific, functional instrument for low back pain. The questionnaire addresses the limitations of daily living by ten specific questions [23].

The SF-36 questionnaire is an unspecific instrument to describe the quality of life in the context of different disease patterns. This instrument is particularly applicable because of its high reliability and the possibility of comparing results to a high number of representative populations. The SF-36 consists of eight scales-rated domains considering the mental and functional outcome. It has been already translated and validated for the German population [24].

### Statistical analysis

Statistical analysis was performed using SPSS (Version 20, 76 Chicago, IL, USA). Data are presented as absolute means and as mean percentage values. Continuous variables were compared using the *t* test in case of approximately normally distributed data. The Chi squared test was used to compare the counts of categorical responses between two independent groups. Analysis of variance (ANOVA) was used to compare between means of more than two groups for data of a normal distribution and homogeneity of variance. Linear correlation between two variables was measured by the use of Pearson’s correlation coefficient. In case of continuous variables, Spearman’s correlation coefficient was used. Statistical significance was defined as *p* < 0.05.

## Results

Between May 2012 and October 2014, a total of 25 patients (SG) with acute fractures of the thoracolumbar junction (T11–L2) and 23 healthy control individuals (CG) were incorporated into the study. The groups (SG and CG) were equal regarding gender distribution (male/female: SG 13/12; CG 10/13; NS), age (SG 50.84 ± 15.43; CG 46.00 ± 13.10; NS) and BMI (SG 26.39 ± 3.52; CG 25.00 ± 4.36; NS). Fifteen patients (60%) were treated by open surgery, while 10 (40%) were treated percutaneously. Predominantly, the vertebral bodies T 12 (6) and L1 (14) were injured, while fractures of T11 (2) and L2 (3) were less frequently diagnosed (Table 1).

**Table 1:** Demographic data

	OS	MIS	CG	
*n*	15	10	23	
Gender (female/male)	8/7	5/5	10/13	NS
Age (years)	51.4 ± 17.66	50.00 ± 12.19	46.00 ± 13.10	NS
BMI ([kg]/[m^2^])	26.05 ± 3.11	26.91 ± 4.18	25.00 ± 4.36	NS
Injured vertebra				
T 11	1	1	–	–
T 12	4	2		
L 1	9	5		
L 2	1	2		

*OS* open surgery; *MIS* minimally invasive surgery; *CG* control group; *BMI* body mass index; *NS* not significant

Additional anterior stabilization was performed in 64% (*n* = 16/25) of all patients, 124 days (± 89 days) after the first surgery. Thirteen of these patients were initially treated by an open dorsal intervention, whereas 3 were treated percutaneously. The anterior stabilization was performed thoracoscopically in all patients with fractures of L1 and above (13 patients) und by a retroperitoneal lumbar approach for fractures below L1 (3 patients).

### Muscle thickness and function of the multifidi muscles

Patients showed 12 month after surgery a slightly larger muscle thickness of the relaxed as well as of the contracted multifidi muscles at level L3/4 compared to the control group; however, this did not reach statistical significance (muscle diameter: relaxed: SG 28.2 ± 5.8 mm; CG 26.5 ± 5.2 mm; NS/contracted: SG 29.1 ± 5.8 mm; CG 27.8 ± 5.1 mm, NS). Similar results were found at level L4/5 (muscle diameter: relaxed: SG 29.9 ± 5.1 mm; CG 28.4 ± 5.2 mm; NS/contracted: SG 30.8 ± 5.1 mm; CG 29.4 ± 5.4 mm; NS) (Table 2).

**Table 2:** Muscle thickness and function of the multifidi and the transverse abdominis muscle

	OS + MIS	CG	
Muscle thickness Multifidus L3/4 (relaxed)	28.2 ± 5.8	26.5 ± 5.2	NS
Muscle thickness Multifidus L3/4 (contracted)	29.1 ± 5.8	27.8 ± 5.1	NS
Muscle thickness Multifidus L4/5 (relaxed)	29.9 ± 5.1	28.4 ± 5.2	NS
Muscle thickness Multifidus L4/5 (contracted)	30.8 ± 5.1	29.4 ± 5.4	NS
VMA multifidus L3/4	3.2%	5.1%	*p* < 0.05
VMA multifidus L4/5	3.1%	3.7%	NS
Muscle thickness Transverse abdominis (relaxed)	4.02 ± 1.4	3.43 ± 0.8	NS
Muscle thickness Transverse abdominis (contracted)	5.08 ± 1.6	4.97 ± 1.1	NS
VMA transverse abdominis	33.43%	37.84%	*p* < 0.05

*OS* open surgery; *MIS* minimally invasive surgery; *CG* control group; *VMA* voluntary muscular activity, *NS* not significant

In contrast, the muscle function of the multifidi muscles at level L3/4 was significantly diminished in the patients compared to the control group (VMA at Level L3/4: SG 3.2%; CG 5.1%; *p* < 0.05). On level L4/5, the muscle function was also reduced in the patient group, but these findings did not reach significant relevance (VMA at Level L4/5: SG 3.1%; CG 3.7%; NS) (Table 2). Regarding the surgical approach (open vs. percutaneous) neither differences in muscle thickness (muscle diameter at L3/4 and L4/5: relaxed: open 28.8 ± 4.7 mm; percutaneous 29.5 ± 5.7 mm; NS/contracted: open 29.6 ± 4.7; percutaneous 30.5 ± 5.7 mm, NS) nor muscle function were observed (VMA L3/4 and L4/5: open 2.8%; percutaneous 3.7%; NS).

### Muscle thickness and function of the transverse abdominis and the oblique abdominal muscles

The abdominal muscles (TrA + EO + IO) did not show a significant difference regarding the muscle diameter between the patients and the control group, during relaxation (overall diameter relaxed: SG 20.09 ± 5.0, CG 20.11 ± 4.7; NS) or during contraction (overall diameter contracted: SG 21.47 ± 5.3, CG 22.21 ± 4.8; NS). Analogous to the multifidi muscles, the function of the transverse abdominis muscle was significantly reduced in the spine patients compared to the control group (VMA TrA: SG 33.43%; CG 37.84%; *p* < 0.05) (Table 2).

Patients treated by the open and the percutaneous approach showed similar results regarding the muscle thickness of the transverse abdominis (Muscle Diameter TrA relaxed: open 4.39, percutaneous 3.46; NS/contracted: open 5.46, percutaneous 4.5; NS). Regarding the muscle function, a trend to a higher amount of intentional contraction in the percutaneous patients was seen, but these results did not reach statistical significance (VMA TrA: open 29%, percutaneous 40%; NS).

Patients treated by stand-alone posterior stabilization and those treated by additional anterior treatment showed equal results considering the muscle size and voluntary activation of the transverse abdominis muscle (muscle diameter TrA: relaxed: stand-alone 4.0 ± 1.6 mm; anterior treatment 4.0 ± 1.4 mm; NS/contracted: stand-alone 5.0 ± 1.7 mm; anterior treatment 5.3 ± 1.6 mm, NS) (VMA TrA: stand-alone 1.0%; anterior treatment 1.3%; NS).

### Clinical outcomes

The VAS spine score revealed that patients still reported substantial pain 1 year after surgery compared to the control group (VAS: SG 46.53 ± 22.60; CG 84.23 ± 20.26; *p* < 0.001) (Table 3).

**Table 3:** Comparison of the clinical outcome between patients and the control

Questionnaire	OS + MIS (*n *= 25)	CG (*n *= 23)	*t* test: *p*
VAS spine score	46.5	84.2	0.000*
ODI	39.7	8.3	0.000*
SF 36			
Physical functioning	46.8	81.1	0.000*
Physical role functioning	17	83	0.000*
Bodily pain	36.5	76.4	0.000*
General health perceptions	51.2	73.4	0.000*
Vitality	43.8	63.9	0.001*
Social role functioning	73.5	90.3	0.005*
Emotional role functioning	52	98.5	0.000*
Mental health	61.1	78.2	0.002*

*OS* open surgery; *MIS* minimally invasive surgery; *CG* control group

*Significant difference between patients and control

The Oswestry Disability Index showed moderate impairments for the spine patients, whereas the control group showed regular results (ODI: SG 39.72% ± 21.35; CG 8.25% ± 14.08; *p* < 0.001) (Table 3).

The physical and mental component summary scales (PCS/MCS) of the SF 36 Questionnaire showed that the health-related quality of life was still limited in patients who underwent posterior stabilization compared to the control group 1 year postoperatively (PCS: SG 31.67; CG 48.44 MCS: SG 47.2; CG 54.52). This was also confirmed by the fact that patients showed significant deficits in all eight sub-qualities of the questionnaire compared to the control group (Table 3).

No correlation between the muscle parameters and the results of the VAS spine score, the ODI or the SF-36 Questionnaire were found (Table 4).

**Table 4:** Correlation between the clinical outcome and the muscle parameters

	VAS spine score	ODI	SF36
Muscle thickness Multifidus L3/4	*r* = − 0.163 NS	*r* = 0.88 NS	*r* = 0.033 NS
Muscle thickness Multifidus L4/5	*r* = 0.005 NS	*r* = 0.023 NS	*r* = 0.033 NS
VMA multifidus L3/4	*r* = 0.176 NS	*r* = − 0.037 NS	*r* = 0.005 NS
VMA multifidus L4/5	*r* = 0.201 NS	*r* = − 0.203 NS	*r* = 0.070 NS
Muscle thickness Transverse abdominis	*r* = − 0.052 NS	*r* = 0.320 NS	*r* = 0.018 NS
VMA Transverse abdominis	*r* = − 0.183 NS	*r* = 0.033 NS	*r* = 0.020 NS

*r* correlation coefficient; *NS* not significant; *VMA* voluntary muscular activity

Nether the VAS spine score nor the ODI and the SF 36 score showed significant differences between patients treated by the open or the percutaneous approach (VAS: open 45.95 ± 24.71; percutaneous 47.40 ± 20.73; NS; ODI: open 43.52% ± 20.92; percutaneous 34.40% ± 21.89; NS; SF-36 PCS: open 32.16; percutaneous 30.89; NS MCS: open 44.57; percutaneous 51.15; NS) (Table 5).

**Table 5:** Comparison of the clinical outcome between open and percutaneously treated patients

Questionnaire	OS (*n *= 15)	MIS (*n *= 10)	*t* test: *p*
VAS WS	46.0	47.4	NS
ODI	43.5	34.4	NS
SF-36			
Physical component summary scale	32.16	30.89	NS
Mental component summary scale	44.57	51.15	NS

*OS* open surgery; *MIS* minimally invasive surgery; *CG* control group; *NS* not significant

## Discussion

The majority of traumatic spine fractures are located in the thoracolumbar junction. Considering the fracture type, surgical treatment is frequently necessary [1, 2, 5, 13–19]. Studies indicate an advantage of the percutaneous technique regarding muscular rehabilitation compared to the conventional open approach [2, 13, 19]. Whether this is beneficial in the context of the clinical outcome has to be answered more sufficiently.

### Strengths and limitations

Our results have to be interpreted with caution, because the statistical strength of our study is limited due to the small number of included patients. Besides, 16 of our patients received an anterior stabilization in the postoperative course. This might have negative impact on the muscular rehabilitation and present inferior results. On the other hand, we believe that the highly accurate performance of the ultrasound investigation represents the main strength of our study and make our results unique and relevant.

In accordance with other studies, our results showed a diminished voluntary muscular function of the multifidus and the transverse abdominis muscle in posteriorly stabilized patients when compared with the control group [25]. These results are confirmed by other studies that found deficits in the muscular function of the multifidus muscles after posterior spinal surgery [13, 14]. Interestingly, these deficits in the muscle function were not associated with a decline in the muscle diameter. This phenomenon can be explained by the presence of intramuscular edema. Stevens et al. revealed that this type of edema still exists 6 month postoperatively [26]. However, other investigations found that this edema disappears within 1 year of follow-up [27]. Besides, other structural alterations of the muscle, such as fatty degeneration, fibrosis and hyperactivation of the muscles [28] should be considered as potential causes for the increased muscle diameter of patients treated by posterior stabilization in our study group. Unfortunately, due to the design of the study, we were unable to further investigate the background of these observed changes in more detail.

In our study, the muscle diameter of the multifidus and the transverse abdominis muscles of trauma patients treated by open and percutaneous interventions was comparable. This is in contrast with a study of Stevens et al. who found less edema in the multifidus muscles of patients treated by a minimally invasive approach compared to open [26]. One explanation for this different finding could be that Stevens examined the edema by MRI at the level of the operated segment. This could be an indicator that the local muscle damage at the segment level is even more pronounced. Fan et al. reports that the minimally invasive approach is associated with less multifidus muscle damage [13], and Cawley et al. even postulates that the minimally invasive approach is superior considering the preservation of the medial branch of the posterior ramus of the spinal nerve compared to the open approach [14]. Although we did not examine the extent of the neurogenic damage within this study, we also found a trend to higher voluntary muscle activation in percutaneously treated patients compared to those treated with open stabilization.

Interestingly, we found no differences in the muscle size and muscle function of patients treated by stand-alone posterior stabilization and those treated by an additional anterior treatment in the course, although especially in the area of the oblique abdominal muscles a deficit could be expected. In our study, this might be explained by the fact that the anterior stabilization was predominantly performed thoracoscopically and just three patients were treated by a retroperitoneal lumbotomy. Chatterjee et al. report that the weakness of the oblique abdominal muscles is common following flank incisions. They observed that almost 50% of patients experience a flank bulge after open nephrectomy [29]. In our study, we also had one patient with a flank bulge following a lumbotomy, but a significant reduced voluntary muscular activation could not be found due to the small number of flank incisions.

Furthermore, it must also be mentioned that anterior treatment within this study was performed 4 months following the posterior stabilization. This course of action could have had a negative impact on the patient’s outcome within this study. Today, we would recommend anterior treatment early after the posterior stabilization to prevent progressive posttraumatic kyphosis. Considering the VAS spine score, we had comparable results between patients treated by open surgery and those treated percutaneously. These findings support results from other series [19, 30] and show that there is no decisive superiority of either of both surgical approaches regarding the postoperative pain, at least 1 year after surgery.

Wang et al. found superior results regarding the ODI at 3 and 6 month after surgery for patients treated minimally invasively. They further describe that this difference gradually diminished over time [19]. We also found slightly better results regarding the ODI for patients treated by the percutaneous approach; however, these differences did not reach statistical significance. Charles et al. found equal results and showed that there was a consistent improvement regarding the activities of daily living over the postoperative course [30].

Within the SF-36 score, we found comparable results between open and percutaneously treated patients. Both groups showed marked impairments regarding the physical and mental status compared to a healthy control group. This is in line with the findings of Wild et al. who performed a 5-year follow-up between patients treated either open or percutaneously [31]. They showed that 5 years after surgery, the results almost achieved normal values. Another study demonstrated that the condition of posterior stabilized patients 1 year postoperatively is comparable to patients with chronic back pain considering the quality of life [30]. This might lead to the assumption that the rehabilitation process of patients treated by posterior stabilization was still far from being completed 1 year after surgery.

Our study showed that no correlation between the muscle function and the clinical outcome, defined as the results of the clinical questionnaires, can be found in patients with posterior stabilizations following traumatic fractures of the thoracolumbar junction 1 year after surgery. This underlines the findings of Wang et al. who postulates that minimally invasive surgery is superior considering the iatrogenic muscle trauma, but this only causes benefits in a short-term period up to 3 months [19]. In contrast, Fan et al. reported even 1 year after surgery less muscle damage in patients treated minimally invasively concomitant with less back pain and less functional disability [13]. Other studies showed that the clinical outcome is also affected by many other existing factors such as the severity of the initial injury [17] or the restoration of the sagittal profile [30, 31]. By means of our study, we think that iatrogenic muscle damage should not be ignored considering the clinical outcome and the rehabilitation of posterior stabilized patients, but this aspect should only play a minor role considering the decision for or against an open treatment.

## Conclusion

The function of the lumbar multifidus and the transverse abdominis muscle is still compromised in patients 1 year after posterior stabilization of traumatic fractures of the thoracolumbar junction. However, muscle deficits did not differ between patients treated either by an open or percutaneous dorsal procedure and muscle function alone seemed not decisive considering the clinical outcome in trauma patients. Therefore, further studies are necessary to investigate the complex interaction of different parameters (e.g., muscle function, sagittal balance) influencing the clinical outcome of dorsally stabilized trauma patients.
